# Reply: Dementia in spontaneous intracranial hypotension: look at the spine

**DOI:** 10.1007/s00234-024-03529-2

**Published:** 2024-12-23

**Authors:** Horst Urbach, Niklas Lützen, Katharina Wolf, Jürgen Beck

**Affiliations:** Department of Neuroradiology, Medical Center – University of Freiburg, Faculty of Medicine, University of Freiburg, Freiburg, Germany

Dear Editor,

We appreciated the authors comment to the term “spinal dementia” but used it intentionally in order to draw the reader’s attention to the spine and to cause her or him to study it adequately [1].

Clinically, it can be difficult to identify patients with behavioral variant frontotemporal brain sagging syndrome (bvFTBSS): In a large series, only 11 of 21 bvFTBSS patients (48%) presented with orthostatic headaches, 16 of 21 patients (76%) had an initial presentation of orthostatic headaches, sometimes years ago [2].

From a pathophysiological point of view, bvFTBSS and behavioral variant frontotemporal lobar degeneration (bvFTLD) are different disease. While the latter is a protein aggregation disorder with TDP43 pathology underpinning approximately 45% and various tauopathies the rest of FTLD cases [3], the pathomechanism in bvFTBSS is unknown. On MRI, bvFTBSS differs from bvFTLD as no frontotemporal atrophy is present but also from “classic” SIH as sagging of the elongated brainstem and of the mesial temporal and basal frontal lobes are more pronounced. A fronto-temporal hypometabolism on PET fits to the symptoms of apathy and disinhibition and on MRI the hippocampi and the basal frontal lobes are clearly sucked towards the interpeduncular cistern (Fig. 1).

**Fig. 1:**
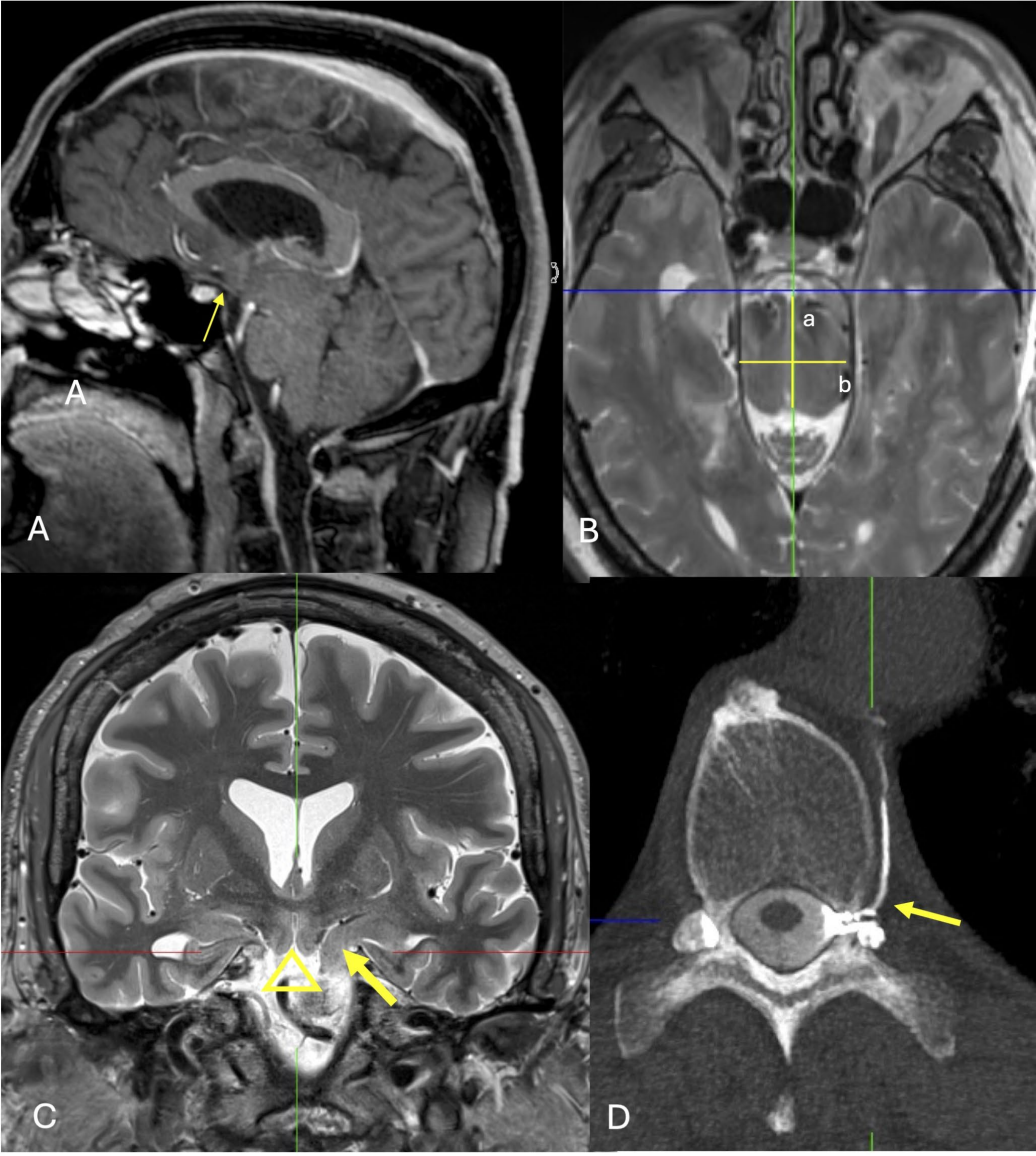
Sagittal contrast-enhanced MPRAGE (**A**), axial T2-weighted SPACE (**B**), coronal T2-weighted STIR images (**C**) in a 56-year-old man with apathy since four years. Note the sucking of the brainstem, of the mesial temporal lobes, of the basal frontal lobes towards the interpeduncular cistern. The optic tract is sucked downwards (A: arrow), the upper brainstem has a longer a.p. diameter (B: a/b), the left hippocampus is sucked over the petroclinoid fold (C: arrow), and the basal frontal lobes point downwards (C: triangle). Dynamic left lateral CT myelography revealed a left-sided CSF venous fistula at Th10/11 (D: arrow). The patient rapidly improved after surgical ligation

Most important for the patients however, is the fact that a spinal CSF venous fistula is found in 1/4 − 1/3 of bvFTBS patients provided patients are studied in lateral decubitus positions [1, 4–8], i.e. the position with the highest yield for detecting a CVF. A ventral spinal CSF leak is found occasionally [6]. A posterior fossa CSF leak causing SIH has been described in a 2-year-old boy [9]. However, that a cranial CSF leak causes SIH is generally questioned or at least very rare [10]. Why (posttraumatic or postsurgical) skull leaks produce SIH, is not really understood [11]. We believe it is mandatory to look for spinal CSF venous fistulas as they can be easily treated with clinical symptomsrapidly improving [7]. In the majority of the bvFTBSS patients however, no CSF leak is found. Fortunately, many of these patients benefit from high-volume non targeted epidural blood patches. Also, spontaneous resolution of bvFTBSS has been described [12]. We described patients who developed a decline in cognitive abilities that affected their ability to perform everyday activities thus fulfilling the definition of dementia [1]. We classified the syndrome dementia not by describing morphological brain changes such as “mesial temporal and parietal atrophy” or “brain sagging” but more specifically by the causal disease, that are e.g. amyloid and tau depositions in Alzheimer’s disease and often a spinal CSF leak in spinal dementia. We also had in mind that most SIH patients have no dementia but many complain of “brain fog” and some of them have mild cognitive impairment as indicated by the trail making test part B (TMT B) which explores executive functions [13–14]. Is it correct to classify a disease by merging the syndrome (here: dementia) and the cause (here: spinal CSF loss) putting the affected organ (here: fronto-temporal and brainstem structures) in the back? Well, many dementia syndromes are classified this way (Alzheimer’s disease, vascular dementia, Lewy Body dementia e.g.). Only, when we do not know the underlying cause exactly, we describe the morphological brain changes. We fully agree that cognitive deficits are not the primary symptoms in infratentorial hemosiderosis patients. Patients typically have progressive ataxia and hearing problems over a long time and eventually pull back from active living [1, 15]. Sometimes, these patients had MRI scans of the head but not of the spine, and the underlying cause of infratentorial hemosiderosis was not recognized. In conclusion, spinal (CSF loss) dementia is an oversimplified term but points to the cause of the disease. The term spinal dementia helps that the root cause is being identified and not the brain sagging or superficial siderosis just described.

## Electronic Supplementary Material

Below is the link to the electronic supplementary material.

Supplementary Material 1

## Data Availability

No datasets were generated or analysed during the current study.
