# Supplementary material for: Reply: Dementia in spontaneous intracranial hypotension: look at the spine
Source: Neuroradiology. 2024 Dec 23;67(1):3–5. doi: 10.1007/s00234-024-03529-2 (PMC11802694; doi:10.1007/s00234-024-03529-2)
Supplement: Supplementary file 1 — Supplementary Material 1 [file 234_2024_3529_MOESM1_ESM.docx]

**Letter to the Editor**

**Dementia in Spontaneous Intracranial Hypotension: A Call for Accurate Terminology**

Dear Editor,

We read with great interest the article by Urbach et al., *“Spinal Dementia: Don’t Miss It, It’s Treatable,”* recently published in Neuroradiology [1]. The paper highlights an underdiagnosed and important phenomenon – dementia caused by spontaneous intracranial hypotension (SIH) – and raises awareness about the potential reversibility of this condition when recognized and treated early. However, we would like to address concerns regarding the terminology used, specifically the term “spinal dementia,” which we believe is both inaccurate and misleading.

While most CSF leaks in SIH patients are indeed located in the spine, cranial leaks leading to brain sagging and behavioral variant frontotemporal dementia (bvFTD)-like signs and symptoms have also been documented [2]. In fact, as seen in one of the five cases described by Urbach et al. [1], a CSF leak is not always identifiable in patients presenting with bvFTD-like symptoms [3, 4]. This raises the possibility that cranial leaks are also underdiagnosed and should not be overlooked. Notably, CSF-venous fistulas, an important cause of bvFTD in SIH patients, were only identified a decade ago [5].

Moreover, it is the CSF leakage per se causing intracranial hypotension and CSF hypovolemia that may result in cognitive and behavioral impairments, independent of the exact location of CSF leakage (spinal or cranial). This is evident by the fact that only few patients with CSF leaks develop bvFTD. In addition, while infratentorial hemosiderosis can lead to cerebellar dysfunction and some degree of cognitive slowing, its clinical presentation differs significantly from the true bvFTD profile or the “brain sagging dementia” previously described in the literature [4].

Raising awareness about SIH and its potential for severe consequences is undoubtedly crucial, and we applaud the authors for their valuable contribution to this effort. However, we believe that the term “spinal dementia” may oversimplify the complex pathomechanism involving both intracranial and spinal dynamics in these cases. The use of precise and accurate terminology will ensure clarity and avoid potential misunderstandings, ultimately supporting better recognition and treatment of this underdiagnosed condition.

Thank you for the opportunity to engage in this important discussion.

References

1.	Urbach H, El Rahal A, Wolf K, Zander C, Demerath T, Volz F, et al. Spinal dementia: Don't miss it, it's treatable. Neuroradiology. 2024;66(10):1671-9.

2.	Lashkarivand A, Eide PK. The first report on brain sagging dementia caused by a cranial leak: A case report. Frontiers in neurology [electronic resource]. 2022;13:1006060.

3.	Schievink WI, Maya MM, Barnard ZR, Moser FG, Jean-Pierre S, Waxman AD, et al. Behavioral Variant Frontotemporal Dementia as a Serious Complication of Spontaneous Intracranial Hypotension. Operative Neurosurgery. 2018;15(5):505-15.

4.	Lashkarivand A, Eide PK. Brain Sagging Dementia -- Diagnosis, Treatment, and Outcome: A Review. Neurology. 2022;25:25.

5.	Schievink WI, Moser FG, Maya M. CSF-venous fistula in spontaneous intracranial hypotension. Neurology. 2014;83(5):472-3.

Urbach H, Lützen N, Wolf K, Beck J

**Reply: Dementia in Spontaneous Intracranial Hypotension: Look at the spine**

^1^Dept. of Neuroradiology, Medical Center – University of Freiburg, Faculty of Medicine, University of Freiburg, Germany

^2^Dept. of Neurosurgery, Medical Center – University of Freiburg, Faculty of Medicine, University of Freiburg, Germany

Corresponding author: Horst Urbach, MD,

Dept. of Neuroradiology, University Medical Center Freiburg,

Breisacher Str. 64, 79106 Freiburg, Germany

Tel.: +49-761-270-51810, Fax: +49-761-270-51950

email: horst.urbach@uniklinik-freiburg.de

ORCID: orcid.org/0000-0001-7264-4807

Dear Editor,

We appreciated the authors comment to the term “spinal dementia” but used it intentionally in order to draw the reader’s attention to the spine and to cause her or him to study it adequately [1].

Clinically, it can be difficult to identify patients with behavioral variant frontotemporal brain sagging syndrome (bvFTBSS): In a large series, only 11 of 21 bvFTBSS patients (48%) presented with orthostatic headaches, 16 of 21 patients (76%) had an initial presentation of orthostatic headaches, sometimes years ago [2].

From a pathophysiological point of view, bvFTBSS and behavioral variant frontotemporal lobar degeneration (bvFTLD) are different disease. While the latter is a protein aggregation disorder with TDP43 pathology underpinning approximately 45% and various tauopathies the rest of FTLD cases [3], the pathomechanism in bvFTBSS is unknown.
On MRI, bvFTBSS differs from bvFTLD as no frontotemporal atrophy is present but also from “classic” SIH as sagging of the elongated brainstem and of the mesial temporal and basal frontal lobes are more pronounced. A fronto-temporal hypometabolism on PET fits to the symptoms of apathy and disinhibition and on MRI the hippocampi and the basal frontal lobes are clearly sucked towards the interpeduncular cistern (Fig. 1).
Most important for the patients however, is the fact that a spinal CSF venous fistula is found in 1/4 - 1/3 of bvFTBS patients provided patients are studied in lateral decubitus positions [1, 4-8], i.e. the position with the highest yield for detecting a CVF. A ventral spinal CSF leak is found occasionally [6]. A posterior fossa CSF leak causing SIH has been described in a 2-year-old boy [9]. However, that a cranial CSF leak causes SIH is generally questioned or at least very rare [10]. In this respect, as long as the location of the skull leak is not reported the case of Lashkarivand and Eide is not convincing [11]. We believe it is mandatory to look for spinal CSF venous fistulas as they can be easily treated with clinical symptoms rapidly improving [7]. In the majority of the bvFTBSS patients however, no CSF leak is found. Fortunately, many of these patients benefit from high-volume non targeted epidural blood patches. Also, spontaneous resolution of bvFTBSS has been described [12].

We described patients who developed a decline in cognitive abilities that affected their ability to perform [everyday activities](https://en.wikipedia.org/wiki/Activities_of_daily_living) thus fulfilling the definition of dementia [1]. We classified the syndrome dementia not by describing morphological brain changes such as “mesial temporal and parietal atrophy” or “brain sagging” but more specifically by the causal disease, that are e. g. amyloid and tau depositions in Alzheimer’s disease and often a spinal CSF leak in spinal dementia. We also had in mind that most SIH patients have no dementia but many complain of “brain fog” and some of them have mild cognitive impairment as indicated by the trail making test part B (TMT B) which explores executive functions [13-14].
Is it correct to classify a disease by merging the syndrome (here: dementia) and the cause (here: spinal CSF loss) putting the affected organ (here: fronto-temporal and brainstem structures) in the back? Well, many dementia syndromes are classified this way (Alzheimer’s disease, vascular dementia, Lewy Body dementia e.g.). Only, when we do not know the underlying cause exactly, we describe the morphological brain changes. We fully agree that cognitive deficits are not the primary symptoms in infratentorial hemosiderosis patients. Patients typically have progressive ataxia and hearing problems over a long time and eventually pull back from active living [1, 15]. Sometimes, these patients had MRI scans of the head but not of the spine, and the underlying cause of infratentorial hemosiderosis was not recognized.

In conclusion, spinal (CSF loss) dementia is an oversimplified term but points to the cause of the disease. The term spinal dementia helps that the root cause is being identified and not the brain sagging or superficial siderosis just described.

References:

1. Urbach H, El Rahal A, Wolf K, et al. (2024) Spinal dementia: Don’t miss it, it’s treatable. Neuroradiology 66:1671-1679
2. Schievink WI, Maya M, Bernard Z, et al. (2022) The reversible impairment of behavioral variant frontotemporal brain sagging syndrome: Challenges and opportunities. Alzheimer’s Dement. 8:e12367
3. Cullinane PW, Wrigley S, Bezerra Parmera J, et al. (2024) Pathology of neurodegenerative disease for the general neurologist. Pract Neurol 24:188–199
4. Luetzen N, Dovi-Akue P, Fung C, Beck J, Urbach H (2021) [Spontaneous intracranial hypotension: diagnostic and therapeutic workup.](https://pubmed.ncbi.nlm.nih.gov/34297176/) Neuroradiology 63:1765-1772
5. Lützen N, Demerath T, Würtemberger U, et al. (2024) Direct Comparison of Digital Subtraction Myelography versus CT-Myelography in Lateral Decubitus Position: Evaluation of Diagnostic Yield for CSF-venous Fistulas. J Neurointerv Surg. 16:1060-1065
6. Schmahmann J, Schievink WI (2024) Compulsive Repetitive Flexion With Breath-Holding in Sagging Brain Syndrome. Neurol Clin Pract 14:e200261
7. Schievink WI, Moser FG, Maya MM (2014) CSF-venous ﬁstula in spontaneous intracranial hypotension. Neurology 83:472–473
8. Lützen N, Beck J, Urbach H (2024) Reversible frontotemporal dementia in spinal cerebrospinal fluid-venous fistula. Dtsch Arztebl Int 121:369
9. Schievink WI, Michael LM, 2nd, Maya M, et al. (2021) Spontaneous Intracranial Hypotension Due to Skull-Base Cerebrospinal Fluid Leak. Ann Neurol. 90:514-516
10. Schievink WI, Schwartz MS, Maya MM, et al. (2012) Lack of causal association between spontaneous intracranial hypotension and cranial cerebrospinal fluid leaks. J Neurosurg. 116:749-54
11. Lashkarivand A, Eide PK (2022) The first report on brain sagging dementia caused by a cranial leak: A case report. Frontiers in neurology [electronic resource]. 13:1006060.
12. Kent L, Butterworth R, Butler C (2019) Lessons of the month 3: Spontaneous resolution of frontotemporal brain sagging syndrome. Clin Medicine 19:336-7
13. Wolf K, Volz F, El Rahal A, et al. Mild cognitive impairment in spontaneous intracranial hypotension (SIH) and its rapid reversal by repair of the spinal CSF-leak. Headache in press
14. Tombaugh T (2004) Trail Making Test A and B: Normative data stratified by age and education. Arch Clin Neuropsychol. 2004;19(2):203-214.
15. El Rahal A, Haupt B, Fung C, et al. (2024) Surgical closure of spinal cerebrospinal fluid leaks improves symptoms in patients with superficial siderosis. Eur J Neurol. 31:e16122

**We received no funding**

A

A

B

C

D

a

b

Fig. 1: Sagittal contrast-enhanced MPRAGE (A), axial T2-weighted SPACE (B), coronal T2-weighted STIR images (C) in a 56-year-old man with apathy since four years.
Note the sucking of the brainstem, of the mesial temporal lobes, of the basal frontal lobes
towards the interpeduncular cistern. The optic tract is sucked downwards (A: arrow),
the upper brainstem has a longer a.p. diameter (B: a/b), the left hippocampus is sucked over the petroclinoid fold (C: arrow), and the basal frontal lobes point downwards (C: triangle). Dynamic left lateral CT myelography revealed a left-sided CSF venous fistula at Th10/11 (D: arrow). The patient rapidly improved after surgical ligation.
